# Construction of a *Plasmodium falciparum* Rab-interactome identifies CK1 and PKA as Rab-effector kinases in malaria parasites

**DOI:** 10.1111/boc.201100081

**Published:** 2011-11-11

**Authors:** Fathia Ben Rached, Carinne Ndjembo-Ezougou, Syama Chandran, Hana Talabani, Hélène Yera, Vrushali Dandavate, Pierre Bourdoncle, Markus Meissner, Utpal Tatu, Gordon Langsley

**Affiliations:** * Institut Cochin, Université Paris DescartesSorbonne Paris Cité, CNRS (UMR 8104), 75014 Paris, France; † Inserm U1016Paris 75014, France; ‡ Université Paris DiderotSorbonne Paris Cité, Paris 75013, France; § Department of Biochemistry, Indian Institute of ScienceBangalore, 560012 Karnataka, India; ¶ Division of Infection and Immunity and Wellcome Centre for Parasitology, Faculty of Biomedical and Life Sciences, University of GlasgowGlasgow, United Kingdom

**Keywords:** Interactom, Kinase, Plasmodium, Rab, Yeast, Ypt

## Abstract

**Background information:**

The pathology causing stages of the human malaria parasite *Plasmodium falciparum* reside within red blood cells that are devoid of any regulated transport system. The parasite, therefore, is entirely responsible for mediating vesicular transport within itself and in the infected erythrocyte cytoplasm, and it does so in part via its family of 11 Rab GTPases. Putative functions have been ascribed to *Plasmodium* Rabs due to their homology with Rabs of yeast, particularly with *Saccharomyces* that has an equivalent number of *rab/ypt* genes and where analyses of Ypt function is well characterized.

**Results:**

Rabs are important regulators of vesicular traffic due to their capacity to recruit specific effectors. In order to identify *P. falciparum* Rab (PfRab) effectors, we first built a Ypt-interactome by exploiting genetic and physical binding data available at the *Saccharomyces* genome database (SGD). We then constructed a PfRab-interactome using putative parasite Rab-effectors identified by homology to Ypt-effectors. We demonstrate its potential by wet-bench testing three predictions; that casein kinase-1 (PfCK1) is a specific Rab5B interacting protein and that the catalytic subunit of cAMP-dependent protein kinase A (PfPKA-C) is a PfRab5A and PfRab7 effector.

**Conclusions:**

The establishment of a shared set of physical Ypt/PfRab-effector proteins sheds light on a core set *Plasmodium* Rab-interactants shared with yeast. The PfRab-interactome should benefit vesicular trafficking studies in malaria parasites. The recruitment of PfCK1 to PfRab5B+ and PfPKA-C to PfRab5A+ and PfRab7+ vesicles, respectively, suggests that PfRab-recruited kinases potentially play a role in early and late endosome function in malaria parasites.

## Introduction

There are more than 225 million cases of malaria each year that result in 800000 deaths (http://www.who.int/malaria/world_malaria_report_2010/en/index.html). One reason that this apicomplexa parasite is such a successful pathogen is that it resides within red bloods cells that are MHC class I and class II negative and so are unable to present parasite-derived peptides to the human immune system. The phylum apicomplexa has a number of medically important pathogens that include *Theileria* and *Babesia* that, such as *Plasmodium,* invade and develop within host red blood cells and subsequently modifies them (Plattner and Soldati-Favre, 2008). Mature erythrocytes do not have a nucleus and indeed, lack regulated vesicular transport. In spite of this, the intracellular parasite is able to secrete antigen and metabolic waste such as lactate and import nutrients, such as glucose and haemoglobin (Langsley et al., 2008). Like yeast, *Plasmodium* has to import and export substances to and from its own organelles (Kats et al., 2008) and across its own parasite plasma membrane. In addition, it also faces the challenge of importing and exporting molecules across the parasitophorous vacuole membrane within which it resides and across the infected host cell plasma membrane (Hanssen et al., 2010; Gruring et al., 2011). Little is known about how the parasite regulates the various secretory and import pathways, but likely candidates to play a key role are Rab GTPases due to their established role in regulating vesicular transport in other eukaryotes (Stenmark, 2009; Goud and Gleeson, 2010).

*Plasmodium* parasites have a family of 11 different Rabs (Quevillon et al., 2003). The number of Rabs present in other apicomplexa vary, with *Theileria* and *Babesia* having just 9, *Cryptosporidium* 8, and *Toxoplasma* 15 (Langsley et al., 2008). The reason why the number of individual Rabs varies between different organisms is unknown. However, recent detailed phylogenetic analysis indicates that Rab11B is restricted to alveolates that include apicomplexa, whereas Rab11A orthologues are widespread in eukaryotes (Agop-Nersesian et al., 2010). Consistently, apicomplexa Rab11B is involved in an alveolate-specific function; namely, the biogenesis of a cytoskeleton-like structure called the Inner Membrane Complex (IMC), whereas Rab11A (Langsley and Chakrabarti, 1996) performs a more classical role in parasite cytokinesis (Agop-Nersesian et al., 2009). The vesicular membrane association of a Rab is due to its C-terminal prenylation at a CC or CXC motif and the *P. falciparum* enzyme (geranylgeranyl transferase-1) responsible for this lipid modification has been described (Chakrabarti et al., 1998). The recycling of vesicles from the donor to acceptor membrane involves a transport protein called rabGDI that binds a Rab in its GDP form, and two *Plasmodium* rabGDIs have been identified (Attal and Langsley, 1996). *Plasmodium,* therefore, appears to possesses the basic machinery associated with recycling of Rab proteins. However, Rabs are important regulators of vesicular traffic due to their capacity to recruit specific effectors (Stenmark, 2009), and to date the only specific *Plasmodium* Rab-effector protein to be described is a myosin light chain called MTIP (Agop-Nersesian et al., 2009). Homology searches using Rab-effectors from higher eukaryotes such as the well-known Rab11 effectors FIP (Horgan and McCaffrey, 2009) have failed to identify orthologues.

Rabs in the yeast *Saccharomyces cerevisiae* are mostly referred to as Ypt proteins and their role in regulating vesicular traffic has been well studied both genetically and biochemically for many years (Segev, 2001). A regularly updated body of data is collated and readily accessible via the *Saccharaomyces* genome database (SGD: http://www.yeastgenome.org). Given that both *S. cerevisiae* and *P. falciparum* are haploid unicellular lower eukaryotes, we decided to exploit the wealth of data on Ypt effectors with the goal of identifying parasite-specific Rab-effectors homologous with yeast. Surprisingly, no Ypt-interactome has been previously reported, so we constructed one and then used it to build a *P. falciparum* Rab (PfRab) interactome. The parasite Rab-interactome allowed us to predict and then demonstrate *in vitro* the physical interaction between PfRab5B and Casein kinase 1 (PfCK1) (Barik et al., 1997), and between PfRab5A and PfRab7 and the catalytic subunit of protein kinase A (PfPKA-C) (Syin et al., 2001). We also provide evidence that *in vivo*, the degree of interaction between PfRab5A and PfRab7 with PfPKA-C changes as the parasite develops to a multinucleated schizont within the red blood cell. These physical interactions suggest a mechanism of recruitment of PfCK1 to PfRab5B+ compartments and PfPKA to PfRab5A+ and PfRab7+ vesicles. In addition to identifying PfPKA as a parasite Rab5A- and Rab7-effector protein, our study suggests a possible role for cAMP-dependent protein kinase activity in regulating PfRab5A- and PfRab7-mediated vesicular transport in malaria parasites.

## Results

### Construction of an Ypt-interactome

**Figure 1:**
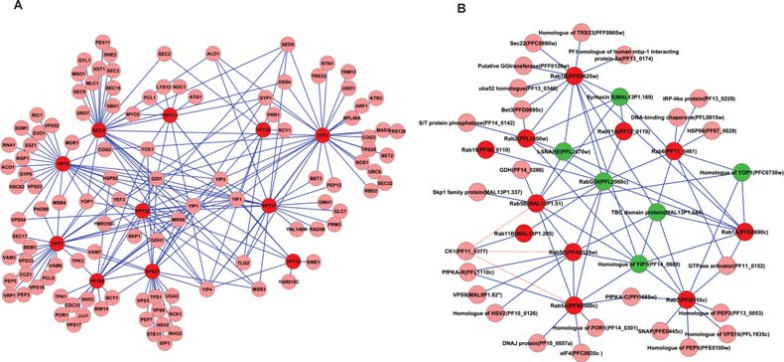
Representation of Physical Ypt/Rab interactions in *S. cerevisiae* and *P. falciparum* (**A**) Physical interactome of the Ypt proteins present in *S. cerevisiae*. (**B**) Physical interactome of PfRab proteins. Red represents Rab proteins and pink indicates the interacting protein. Green indicates effector proteins that physically interact with three or more Ypt/Rab proteins. The putative interaction between PfCK1 and the three PfRab5s is shown in a pink dotted line due to difficulties in assigning a specific PfRab5 isoform.

All interactants catalogued at SGD as having been demonstrated to either genetically, or physically interact with a given Ypt protein were downloaded and an interactome built for the whole Ypt family and for each individual Ypt protein (see Supplementary Table S1 for all Ypt-interactants). As the global (genetic plus physical) Ypt-interactome is complicated to a point of being visibly difficult to interpret it is not shown, and Figure 1A shows only the interactome based on physical interactions with a given Ypt.

**Figure 2:**
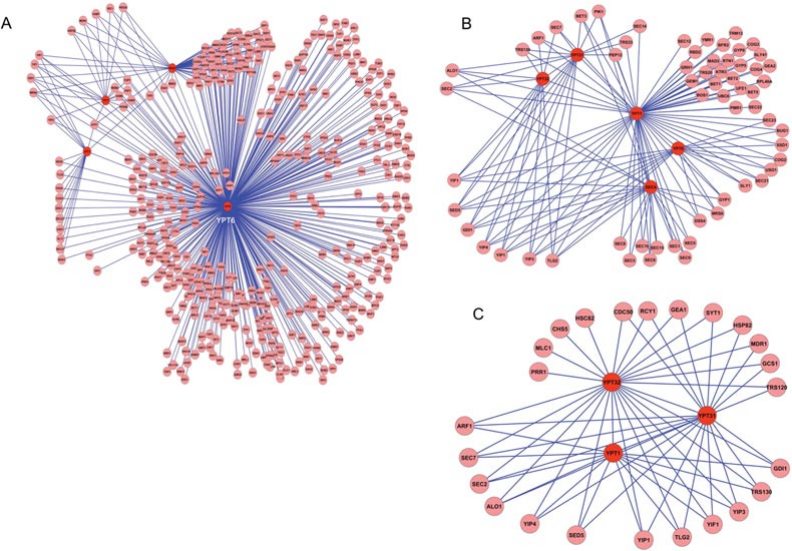
Specific Ypt-interactomes based on all genetic and physical interactions (**A**) Complex interacting network of YPT6, which has more than 100 interacting proteins that include YPT7, YPT1 and VPS21. (**B**) Interactome network of YPT1. (**C**) Representation of a simpler network that shows interacting proteins of YPT32. Red represents Rab proteins and pink indicates the interacting protein.

Specific Ypt-interactomes based on all genetic and physical interactions are shown in Figure 2. The Ypt6 network (Figure 2A) displays a remarkable complexity of interactions that clearly underscores the central role that Ypt6 plays in regulating vesicular transport in yeast. However, not all Ypt proteins display the same level of interactions with Ypt1 having a less complicated network (Figure 2B) and that of Ypt32 being even more reduced (Figure 2C). Individual physical interactomes for six other Ypt proteins are displayed in Supplementary Figure S1. As the range of potential processes involving a given Ypt/Rab is determined in part by the panoply of its specific interactants, it implies that Ypt10 and Ypt11 (Supplementary Figures S1B and S1C) regulate a restricted number of events, in contrast to Ypt1 (Supplementary Figures S1A and S1D) that has the capacity to mediate many.

### PfRab homology and orthology assignments

Multiple reciprocal BLAST analyses has given rise to the PfRab nomenclature (Quevillon et al., 2003) and identified the corresponding homologue in *S. cerevisiae* (Table 1 and Supplementary Figure S2). However, many new Rab sequences from different organisms have since been deposited into public databases and at OrthoMCL DB (http://orthomcl.org) collated into orthology groups. Interrogation of OrthoMCL allows one to identify the orthology group for given PfRab (Table 2). Certain orthology groups are restricted, such as the Rab11B group (OG4_21991) that contains only alveolates, as already mentioned (Agop-Nersesian et al., 2010). PfRab5A belongs to the most restricted orthology group (OG4_36791) that only contains apicomplexa parasites (*Plasmodia, Babesia* and *Theileria*) known to invade erythrocytes. The PfRab5B/Ypt52 orthology group (OG4_18709) is less restricted containing six species, whereas the PfRab5C/Ypt51 (OG4_10168) orthology group is the largest. We built PfRab-interactomes based exclusively on Ypt-Rab pairs that belong to the same orthology group (Table 2). However, for the three PfRab5s we constructed a single interactome, since sequence similarities between these and the yeast Rab5 isoforms did not allow individual isoform-specific orthology assignments to be made with confidence.

**Table 1:** *Plasmodium falciparum* homologues of *S. cerevisiae* Ypt/Rabs

Serial no.	*S. cerevisiae* protein	*P. falciparum* protein
1	SEC4/SRO6	PfRab1A (PFE0690c)
2	YPT1/YP2	PfRab1B (PFE0625w)
3	YPT10	PfRab18 (PF08_0110)
4	YPT11	PfRab11B (MAL13P1.205)
5	YPT31/YPT8	PfRab2 (PFL1500w)
6	YPT32	PfRab11A (PF13_0119)
7	YPT51/Vps21	PfRab5C (PFA0335w)
8	YPT52	PfRab5B (MAL13P1.51)
9	YPT53	PfRab5A (PFB0500c)
10	YPT6	PfRab6 (PF11_0461)
11	YPT7/VAM4/AST4	PfRab7 (PFI0155c)

*Saccharomyces cerevisiae* Ypt amino acid sequences were used to query the PlasmoDB ((http://plasmodb.org/plasmo) and the *P. falciparum* Rab displaying the highest similarity was taken and used to query the SGD data base (http://www.yeastgenome.org). The homology assignment was based on the same Ypt/PfRab pair being identified by such reciprocal BLAST analysis.

**Table 2:** Orthology groups for Rab and Ypt proteins in *P. falciparum* and *S. cerevisiae*

OrthoGroup	PfRab	*S. cerevisiae*	*P. falciparum*	PlasmoDB	SGD	SGD	Ypt	Ypt
OG4_21598	Rab1A	0	1	PFE0690c				
OG4_10503	Rab1B	1	1	PFE0625w	YFL038C		Ypt1	
OG4_11361	Rab2	0	1	PFL1500w				
OG4_36791	Rab5A	0	1	PFB0500c				
OG4_18709	Rab5B	1	1	MAL13P1.51	YKR014C		Ypt52	
OG4_10168	Rab5C	2	1	PFA0335w	YNL093W	YOR089C	Ypt53	Ypt51
OG4_10702	Rab6	1	1	PF11_0461	YLR262C		Ypt6	
OG4_11035	Rab7	1	1	PFI0155c	YML001W		Ypt7	
OG4_10424	Rab11A	2	1	PF13_0119	YGL210W	YER031C	Ypt32	Ypt31
OG4_21991	Rab11B	0	1	MAL13P1.205				
OG4_13079	Rab18	0	1	PF08_0110				
OG4_10784		1			YFL005W		Sec4	
OG4_60929		1					Ypt10	
OG4_61055		1					Ypt11	

Orthologue groups were obtained from OrthoMCL DB (http://www.orthomcl.org/cgi-bin/OrthoMclWeb.cgi) and due sequence similarities certain Ypts/PfRabs appear in the same orthology group.

### PfRab-interactomes

**Figure 3:**
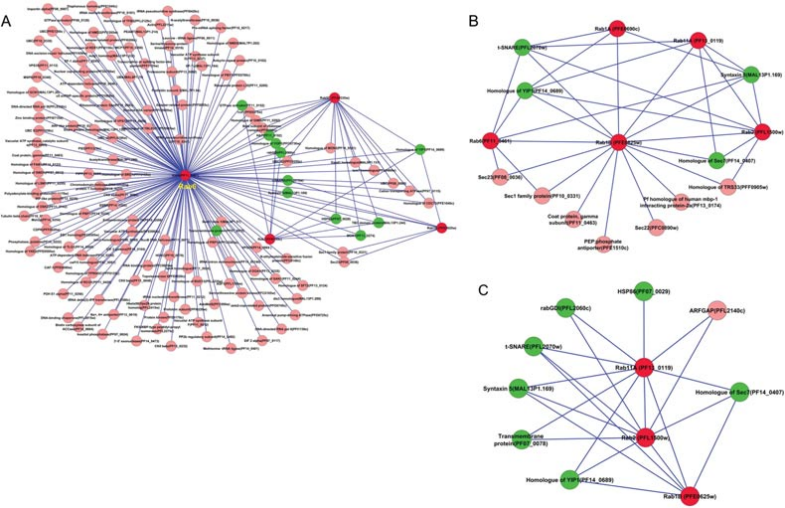
Specific PfRab-interactomes based on all genetic and physical interactions Interactomes of homologues of YPT6, YPT1 and YPT32 that belong to same orthology group. (**A**) Interactome of PfRab6 and similarly it represents a complex interactome like YPT6. (**B**) Interactome of PfRab1B and (**C**) that of PfRab11A. Red represents Rab proteins and pink indicates the interacting protein. Green indicates the proteins that interact with three or more Rab proteins.

We show (Figure 1B) the global physical interactome for the PfRab family that will be discussed in detail below (Supplementary Table S2 lists all predicted genetic and physical PfRab-interactants). For comparison with Figure 2, we present the PfRab6, PfRab1B and PfRab11A interactomes (Figure 3). Like Ypt6, parasite Rab6 is involved in many interactions (Figure 3A) and the homology between Ypt6/PfRab6 effectors underscores the conserved role for this Golgi-specific Rab. There is less conservation between the number of effectors for PfRab1B (Figure 3B) and PfRab11A (Figure 3C), but similar to their yeast orthologues they also show interactomes of reduced complexity.

### PfCK1 is a PfRab5B interactant

**Figure 4:**
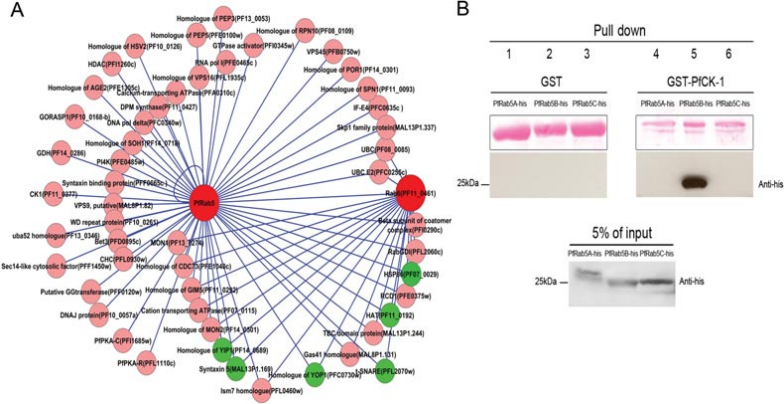
Interactomes of PfRab5 isoforms based on genetic and physical interactions (**A**) The interactome of all three PfRab5 isoforms. Red represents Rab proteins and pink indicates the interacting protein. Green indicates the proteins that interact with three or more Rab proteins. (**B**) Specific physical association between PfRab5B and PfCK1. GST or GST-PfCK1 was incubated in solution with PfRab5A-His or PfRab5B-His or PfRab5C-His recombinant proteins. After washing steps, physical association was revealed by a Western blot with anti-His antibodies. In (B), the amount of input His-tagged Rab protein revealed by anti-His antibodies is given at bottom. In (B), top left shows the amount of GST-only and GST-PfCK1 proteins used as bait and Middle shows that PfRab5B-His binds only to GST-PfCK1 and not to GST, whereas neither PfRab5A nor PfRab5C bind to GST-PfCK1, or GST.

Since both *P. falciparum* and *S. cerevisiae* have three closely related *rab5* genes, we constructed a global Rab5-interactome that illustrates all potential PfRab5-interactions (Figure 4A). There is a myriad of predicted interactants, with a significant number shared with PfRab6 (PF11_0461) (de Castro et al., 1996). Among the specific Rab5-effector proteins, we choose to wet-bench test the physical association predicted with CK1 (PF11_0377, (Barik et al., 1997)) and tested all three His-tagged PfRab5 isoforms for GST-PfCK1 binding in a series of pull-down experiments. In solution and in the presence of 0.4% Triton (see Materials and methods), PfCK1 binds only to PfRab5B (Figure 4B) demonstrating that in spite of their shared sequence identity each PfRab5 isoform can have specific effector proteins.

### Ypt7 versus PfRab7 interactomes

**Figure 5:**
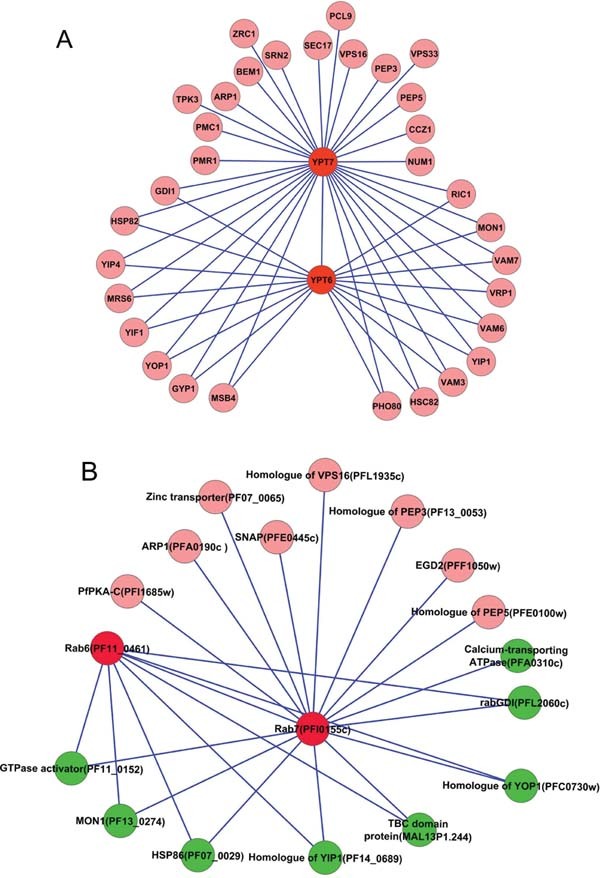
Comparison of interactomes of YPT7 and PfRab7 based on physical interactions (**A**) The interactome of YPT7 and (**B**) that of PfRab7. Red represents Rab proteins and pink indicates the interacting protein. Green indicates the proteins that interact with three or more Rab proteins.

Given that *P. falciparum* is a strict intracellular pathogen that resides for long periods within erythrocytes, additional Rabs involved in regulating endocytic processes is of particular interest. In *S. cerevisiae* one of Ypt7 functions is to regulate homotypic fusion of partner vacuoles, a required step during vacuole inheritance (Haas et al., 1995) and for these reasons we compare in Figure 5 the physical interactomes of Ypt7 and PfRab7. One can immediately see that Ypt7/PfRab7 share a number of effector proteins with Ypt6/PfRab6 implying that these are involved in activities common to both Rabs. Among this group are Hsp82 (YPL240C) and Hsp86 (PF07_0029), and via this interaction PfRab6 and PfRab7 would connect to the Hsp86 network of interactions (Pavithra et al., 2007). With a specific role for PfRab7 in mind the subset of specific effectors are pertinent and here, we note the predicted physical interaction between TPK3 and Ypt7. Our attention was drawn to testing parasite PKA by the recent report that extracellular adenosine signalling in sickle cell red blood cells activates erythrocyte PKA, combined with the observation that ATP signalling via the P2X7 receptor leads to the membrane assembly of actin (Kuehnel et al., 2009a, b; Zhang et al., 2010). Purinergic receptors have been described both at the plasma membrane of *P. falciparum*-infected erythrocytes (Tanneur et al., 2006) and on the parasite's own plasma membrane (Levano-Garcia et al., 2010).

### PfPKA-C is a PfRab7-interactant

**Figure 6:**
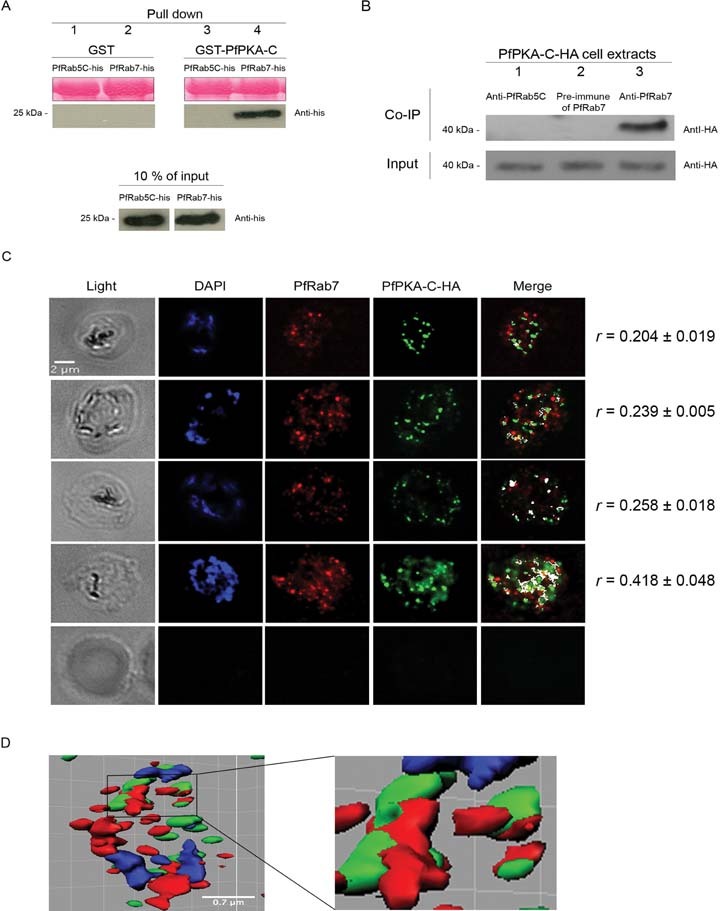
Physical association between PfRab7 and PfPKA-C *in vitro* and *in vivo* (**A**) PfPKA-C specifically binds in solution to PfRab7, but not to PfRab5C. Top, a Ponceau stain showing the amount of GST and GST-PfPKA-C proteins used as bait. Middle, PfRab7 and not PfRab5C binds to GST-PfPKA-C and neither bind to GST-alone. Bottom, indicates the amount of input His-tagged Rab protein, as estimated with anti-His antibodies. (**B**) Top, the presence of PfPKA-C-HA in the anti-PfRab7 immunoprecipitate is revealed with an anti-HA antibody. PfPKA-C-HA was not detected when pre-immune anti-PfRab7 serum, or anti-PfRab5C serum were used. Bottom, equivalent amounts of parasite protein extracts were used for precipitations as revealed by reaction with anti-HA antibodies. (**C**) Association between PfRab7 and PfPKA-C-HA *in vivo* is shown at a single stack of image acquisition. The distribution of PfRab7 (red) is revealed with a specific anti-peptide PfRab7 antibody and the distribution of PfPKA-C-HA (green) is revealed with anti-HA antibodies. The association between PfRab7 and PfPKA-C-HA is shown in white. The Pearson's coefficient (*r*) represents the degree of co-localization and the mean value was calculated by analysing all z-stacks from three independent cells. The level of association between Rab7 and PfPKA-C-HA increases as the parasite develops into a multi-nucleated schizont (nuclei shown stained in blue with DAPI). (**D**) An isosurface 3D-reconstruction with PfRab7 (red) and PfPKA-C-HA (green).

The Ypt7/PfRab7 interactomes suggest the possibility that in *P. falciparum* parasites PfPKA-C (Syin et al., 2001) could be recruited to PfRab7+ vesicles/organelles. Moreover, in *S. cerevisiae* the interactions with the different TPKs are physical and the validity of prediction for *P. falciparum* can again be tested directly. To this end, we exploited the *P. falciparum* transgenic line that expresses the PfPKA-C subunit C-terminally tagged with Hemagglutinin (HA) (Leykauf et al., 2010), and assayed for a physical association with PfRab7 both *in vitro* and *in vivo* (Figure 6). In a series of *in vitro* pull-down experiments using GST-tagged PfPKA-C and either His-tagged PfRab7 or His-tagged PfRab5C, we demonstrated that PfPKA-C physically binds PfRab7 in solution and does not bind PfRab5C (Figure 6A).

Next, we immunoprecipitated from parasite-infected red blood cells both PfRab5C and PfRab7 with specific antibodies and additionally took pre-immune serum to PfRab7 as a negative control. Equivalent amounts of input protein in the cell extracts were verified with the anti-HA antibody (see Materials and methods for details). The three precipitates were transferred to membrane and probed with an anti-HA antibody and PfPKA-C-HA was only detected associated with PfRab7 *in vivo* (Figure 6B).

We further verified an *in vivo* association between PfRab7 and PfPKA-C-HA in parasites by indirect immunofluorescence and one can observe a subset of PfRab7+ vesicles associated with a subset of PfPKA-C-HA+ structures (Figures 6C and 6D). *In vivo,* the association between PfPKA-C and PfRab7 appears to be dynamic with the degree of co-localization (shown in white) increasing (*r* values go from 0.204 to 0.418), as the parasite develops from trophozoites (2–3 nuclei stained blue by DAPI) to a multi-nucleated schizont within the red blood cell (Figure 6C and see video Supplementary Figure 3). Non-infected red blood cells failed to stain (bottom panel). An isosurface 3D reconstruction is presented in Figure 6D, together with an enlargement of the region shown boxed. One can clearly see the close juxtaposition of PfRab7 (red) with PfPKA-C (green).

### Common effectors shared between PfRab5 and PfRab7

**Figure 7:**
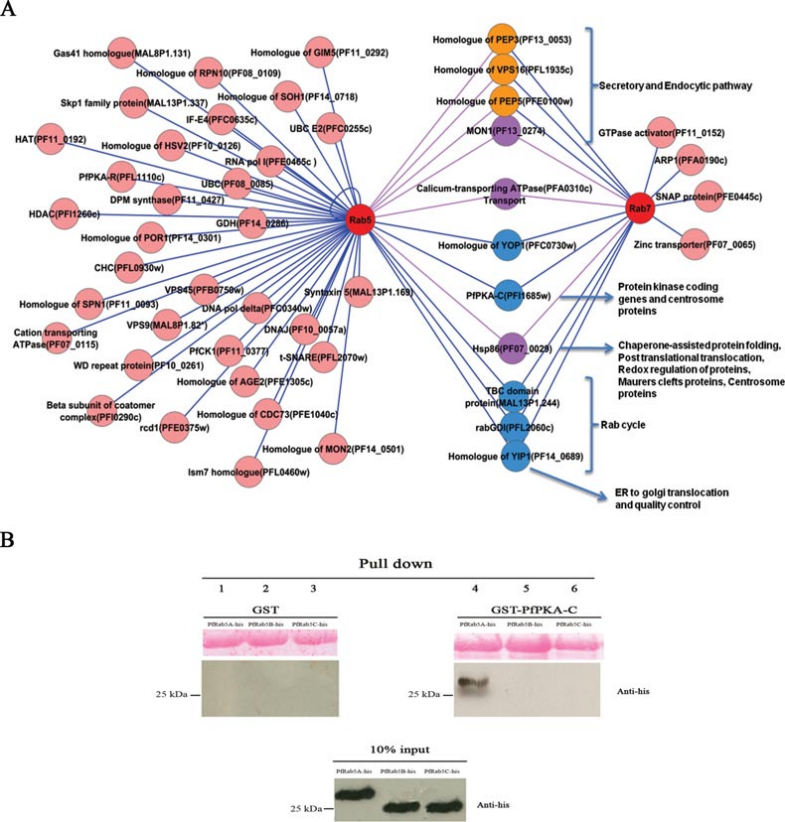
PfRab5 and PfRab7 shared effectors based on predicted genetic and physical interactions (**A**) Blue indicates putative physical effectors shared by Rab5 and Rab7 and purple indicates putative genetic effectors shared by Rab5 and Rab7. Out of the shared effectors, the ones that have a putative physical interaction with Rab7 and genetic interaction with Rab5 are indicated in orange. Potential functional pathways have been indicated for Rab5 and Rab7 common effectors. Pink indicates all other putative effectors predicted to specifically interact with only PfRab5, or PfRab7 that are shown in red. (**B**) Specific physical association between PfRab5A and PfPKA-C. GST-only or GST-PfPKA-C was incubated in solution with PfRab5A-His or PfRab5B-His or PfRab5C-His recombinant proteins. After washing steps, physical association was revealed by a Western blot and the amount of input and bound His-tagged Rab protein revealed by anti-His antibodies. Ponceau staining indicates the amount of GST-only and GST-PfPKA-C proteins used as bait. PfRab5A-His only binds to GST-PfPKA-C and not to GST-alone, whereas neither PfRab5B, nor PfRab5C bind to GST-PfPKA-C, nor GST-alone.

To highlight interactants potentially shared between PfRab5 and PfRab7, we constructed a joint interactome based on both genetic and physical predicted interactions (Figure 7A). The common effectors are colour-coded to distinguish between putative physical (blue) and genetic (purple), and mixed physical (PfRab7) and genetic (PfRab5) interactions are indicated in orange. Among the putative physical effectors shared by PfRab5 and PfRab7 are proteins known to be involved in cycling of Rabs from donor to acceptor compartments. In addition, one can see that PfPKA-C is predicted to bind not only to PfRab7 (see above), but also to a PfRab5 isoform. To identify the PfPKA-C-binding PfRab5 isoform, we again performed pull downs and observed that it binds specifically to PfRab5A and not to PfRab5B, or PfRab5C (Figure 7B).

## Discussion

The purpose of this comparative Ypt- versus PfRab-interactome study was to identify a set of conserved (between yeast and *Plasmodium*) proteins that would represent a novel collection of putative PfRab-interactants. Our analysis of the Ypt6 network shows that it displays a remarkable complexity of interactions that clearly underscores the central role that Ypt6 plays in regulating vesicular transport in yeast. It is immediately obvious that like Ypt6, PfRab6 is involved in many interactions highlighting the conserved role for this Golgi-specific GTPase. Furthermore, individual network complexity is compounded by interactions between Ypt6 with Ypt1, Ypt7 and Ypt52/Vps21 that consequently gives rise to interconnecting networks. There is somewhat less conservation between the number of effectors for PfRab1B and PfRab11A, but similar to their yeast orthologues they also show interactomes of reduced complexity. Clearly, there is a correlation between orthology group size for a given Ypt/Rab and the complexity of the corresponding conserved interactome.

We concentrated on predicted physical Rab-interactions, as these could be directly verified experimentally, since testing genetic interactions in *Plasmodium* parasites is not feasible at this time. To underscore the validity of our approach, we experimentally tested and confirmed three predicted interactions, two for a specific PfRab5 isoform, and one for PfRab7. We provided experimental evidence that supports the prediction that PfCK1 is a PfRab5 effector kinase by demonstrating that PfCK1 binds specifically only to PfRab5B (Figure 4B). The subcellular distribution of PfCK1 has not yet been described (Barik et al., 1997), but in the related apicomplexa *Toxoplasma gondii* there are two CK1 isoforms with TgCK1-alpha being cytosolic and TgCK1-beta being associated via its C-terminus with the plasma membrane (Donald et al., 2005). Our close inspection of TgCK1-beta identified two potential palmitoylation sites in this region (not shown), but whether PfRab5B is involved in trafficking PfCK1 just to the cytosol, or also to the parasite's plasma membrane remains to be determined. We also demonstrated that PfRab5A binds PfPKA-C and shares this effector kinase with PfRab7. Under the conditions used, the shared sequence identity between the three PfRab5 isoforms did not prevent specific binding of PfCK1 to PfRab5B, or PfPKA-C to PfRab5A.

In *T. gondii,* TgRab7 has recently been described associated with a novel vacuolar organelle (Miranda et al., 2010; Parussini et al., 2010). Whether the Rab7+ positive vesicular-like structures identified here in *P. falciparum* correspond to this novel vacuolar organelle remains to be seen. In *P. falciparum* only a subset of vesicles, the number of which increased as the parasite developed into a multinucleated schizont, were found to interact with PfPKA-C suggesting different, dynamic, sub-populations of PfRab7+ vesicles *in vivo*. We also observed PfPKA-C+ structures that were negative for PfRab7 and some of these might be associated with PfRab5A+ vesicles, since we failed to observe any interaction with PfRab5B and PfRab5C *in vitro* or *in vivo* (Figures 6 and 7).

The genome of *S. cerevisiae* encodes three PKA-C (TPK) and one PKA-R (BCY1) subunits with BCY1 being structurally and functionally similar to the mammalian RII class of PKA regulators. *P. falciparum* has only a single regulatory subunit PfPKA-R (Merckx et al., 2008), and a single catalytic subunit PfPKA-C (Syin et al., 2001). TPK1, TPK3 and BCY1 all physically interact with one of the yeast Rab5s (see Supplementary Figure 1F). Consequently, PfPKA-C and PfPKA-R are predicted to physically interact with one of the parasite's PfRab5s (Figures 4 and 7), in addition to PfRab7 (Figure 5). However as stated above, only PfRab5A bound PPfKA-C and whether PfRab5B and/or PfRab5C bind to PfPKA-R will be tested in the future.

The ability of PfRab5A and PfRab7 to bind PfPKA-C suggests that they act somewhat like a PKA-anchor proteins (AKAP), recruiting PKA to early and late endosomes (Beene and Scott, 2007). By using parasite Rabs as pseudo-AKAPs *P. falciparum* may compensate for the paucity of recognizable AKAPs encoded in its genome, as our bioinformatic searches reveal the presence of only a single AKAP-like protein (PFE0640w) showing similarity to AKAP18 (Gold et al., 2008). Nonetheless, it is interesting to note that mammalian Rab4 and Rab11A have been described to bind D-AKAP2 thereby recruiting PKA to recycling endosomes and altering transferrin receptor turnover (Eggers et al., 2009). It would seem then, that like yeast, malaria parasites have a more direct way of recruiting PKA to endosome/vacuole-like structures. It is of course intriguing as to the potential role PKA might be playing on PfRab5A+ and PfRab7+ structures. An exciting possibility is that PKA might be involved in actin mobilization at the endosomal membrane (Kuehnel et al., 2009a, b) and in such a way regulate the parasite's ability to import nutrient (Elliott et al., 2008; Lazarus et al., 2008) and recycle receptors.

## Materials and methods

### Rab-interaction data

*Saccharomyces cerevisiae* was used as the source organism to study PfRab interactors, as its Ypt protein and effectors are characterized and also because it shares the same number of Rab proteins as *Plasmodium falciparum* (Quevillon et al., 2003). The yeast Ypt/Rab genome sequences as well as the physical and genetic interactors were downloaded from SGD database (http://www.yeastgenome.org). Eight hundred twelve interactions involving 596 proteins were found to be involved in the *S. cerevisiae* Ypt/Rab-interactome network. All the redundant interactions were removed and the remaining interactions represented as networks using Cytoscape (http://www.cytoscape.org/).

### Orthologous protein data for PfRab-interactants

The orthologous protein information was obtained by performing pairwise alignment of the sequences using BLASTp at NCBI (http://blast.ncbi.nlm.nih.gov/Blast.cgi). Orthologue groups were obtained from OrthoMCL DB (http://www.orthomcl.org/cgi-bin/OrthoMclWeb.cgi). BLASTp (E-value≤0.05) was performed by taking *S. cerevisiae* protein sequences as queries against *P. falciparum* protein sequences obtained from PlasmoDB (http://plasmodb.org/plasmo) and confirming the hit by reverse BLAST. The first hit obtained was taken as query for the reverse BLAST against non-redundant (nr) protein database with *S. cerevisiae* as organism. Homologues were obtained for 200 proteins.

### PfRab and Rab-interactor homologues

We used BLASTp searches to identify the homologues of *S. cerevisiae* Ypt proteins and their effectors in *P. falciparum*. For those proteins where reverse BLASTp failed to identify a clear homologue due to the best hit being already assigned, the second best hit was taken as the homologue. Due to a high degree of similarity between Ypt10 and Ypt11, multiple sequence alignments (ClustalW) (http://www.ebi.ac.uk/Tools/clustalw2/index.html) were applied together with PfRab11B and PfRab18.

### Constructs for expression of recombinant proteins

To make C-terminal PfRab7-His, PfRab5A-His, PfRab5B-His and PfRab5C-His constructs for expression in *Escherichia coli*, the CDS of *Pfrab7* (PFI0155c), *Pfrab5a* (PFB0500c), *Pfrab5b* (MAL13P1.51) and *Pfrab5c* (PFA0335w) were PCR amplified from cDNA of *P. falciparum* (3D7) and cloned into BamHI/XhoI site of the *E. coli* expression vector pET-21d (Novagen, EMD4Biosciences). The sequences of all expression constructs were verified. To make the N-terminal GST-PfPKA-C construct, a codon optimized synthetic *Pfpka-c* gene (PFI1685w) was custom made (Eurobio) in order to improve recombinant protein levels when expressed in *E. coli.*

### Protein expression in *E. coli*

All constructs were transformed into BL21-CodonPlus (DE3)^−^ RIL strain (Stratagene). LB media contained 34 μg/ml chloramphenicol and 100 μg/ml ampicillin and cells were grown at 37°C to an absorbance at 600 nm. Proteins expression were induced by adding 0.2 mM IPTG for PfRab5A-His, PfRab5B-His, PfRab5C-His and PfRab7-His and 1mM IPTG for GST-PfPKA-C, GST-PfCK1 and GST-alone, and the cultures were incubated overnight at 20°C. Cells were harvested by centrifugation at 5000× *g* for 20 min. Harvested cells were resuspended in urea 6 M buffer supplemented with protease inhibitor cocktail (Roche) for PfRab proteins and in PBS 1X, 1% Triton 100X, 1 mM EDTA buffer supplemented with protease inhibitor cocktail (Roche) for GST-PfPKA-C, GST-PfCK1 and GST-alone, then stored at −80°C. His-tagged proteins were purified on Ni-NTA agarose (Qiagen) in the case of PfRab5A, PfRab5B, PfRab5C and PfRab7, and on Glutathione Sepharose TM 4B beads (GE Healthcare) in case of GST-tagged proteins.

### GST pull downs and western blotting

For GST pull-down experiments, Glutathione Sepharose TM 4B (GE Healthcare) coupled to GST or GST-PfPKA-C or GST-PfCK1 (approximately 2–3 μg of proteins coupled) were incubated with 10 μg of PfRab7-His or PfRab5A-His or PfRab5B-His or PfRab5C-His recombinant proteins overnight at 4°C. These samples were washed four times in PBS (0.4% Triton X-100) and the proteins were eluted by boiling in Laemmli sample buffer and separated by electrophoresis in a 15% SDS-polyacrylamide gel and transferred into nitrocellulose membrane. The blots were first incubated with the mouse monoclonal anti-histidine (1:1000, Covance) and then incubated with the anti-mouse peroxidase-conjugated secondary antibody (1:5000, Sigma Aldrich). Immunoblots were developed by chemiluminescence using ECL (Pierce).

### Cells lines

The *P. falciparum* 3D7 transgenic line expressing PfPKA-C-HA (Leykauf et al., 2010) was cultured in human 0^+^ erythrocytes and positive selection of PfPKA-C-HA expressing parasites was achieved using 10nM WR99210, an anti-folate that selects for the presence of the human *dhfr* gene (de Koning-Ward et al., 2000).

### PfRab-specific antibodies

To generate the PfRab5C-specific antibody, PfRab5C-His protein was purified (see section: *Protein expression in E. coli* section) and used to immunize two rabbits (Eurogentec). To generate specific PfRab7 antibodies, two peptides (FALNNQSEQKMYKSR and FLIQASPKDPENFPF) were synthesized and used to immunize two rats (Eurogentec).

### Co-immunoprecipitation and western blotting

Thirty millilitre of parasite culture at 10% of parasitemia (mostly schizonts) expressing PfPKA-C-HA were saponin lysed. The resulting parasite pellet was washed in ice-cold PBS and lysed in ten volumes of ice-cold RIPA (Euromedex) buffer complemented with protease inhibitor cocktail (Roche). The membrane fraction was separated from soluble proteins by centrifugation at 13000× *g* for 30 min. Protein concentration was evaluated in the resulting supernatant by the Bradford method (Biorad). Five hundred microgram of protein extract was incubated for two h at 4°C with the rat pre-immune serum, anti-PfRab7 antibodies (a mix of both anti-peptide antibodies), or with rabbit anti-PfRab5C antibody, all at 50 μg/ml (Bradford estimation). Thirty microliter of protein-G sepharose beads (GE healthcare) was added to precipitate the complexes and incubated overnight at 4°C. Protein-G sepharose beads were washed four times with ten volumes of cold RIPA buffer. Proteins were eluted by boiling in Laemmli sample buffer and separated by electrophoresis through a 15% SDS-polyacrylamide gel and transferred into nitrocellulose membrane. The blot was incubated with the anti-HA rat antibody (1:1000, Roche) and then incubated with the peroxidase-conjugated anti-rat secondary antibody (1:5000). Immunoblots were developed by chemiluminescence using ECL (Pierce).

### Immunofluorescence

Smears of red blood cells infected with *P. falciparum* expressing PfPKA-C-HA were fixed using 100% cold methanol for 5 min. Cells were washed with PBS, and were permeabilized with 0.1% Triton 100X in PBS for 5 min. After washing with PBS, slides were blocked in 3% BSA PBS for 1 h at room temperature. The slides were incubated successively for 1 h with first antibodies: rat anti-PfRab7 (1:300) and mouse anti-HA (1:200, Sigma Aldrich). The slides were washed four times and incubated with AlexaFluor 594 anti-rat IgG antibodies (1:3000, Molecular Probes Probes), with AlexaFluor 488 anti-mouse IgG antibodies (1:3000, Molecular Probes Probes), with DAPI (1 μg/ml) and then washed and mounted (Dako). Cells were examined under a microscope (Leica DMI 6000, X 100 objective, NA 1.4 oil) with a cooled charge-coupled device camera (Micromax). Images were acquired with z stacks with MetaMorph (Universal Imaging) and de-convoluted with Huygens (SVI). Images were analysed and processed with ImageJ (NIH) and Photoshop (Adobe Systems Inc.). For the merge, the ImageJ co-localization plug-in was used. Pearson's coefficient of co-localization was attributed with the ImageJ JACoP plug-in (Bolte and Cordelieres, 2006). Using immunofluorescence images corresponding to the different z stacks (of the cell in upper middle panel of Figure 6C), a volumic (Supplementary Figure 3) and isosurface (Figure 6D) 3D-modelizations were performed with Imaris (Bitplane).

## Author contribution

Syama Chandran and Vrushali Dandavate performed the BLAST analysis and constructed the yeast and parasite Rab interactomes. Fathia Ben Rached, Carinne Ndjembo-Ezougou and Hélène Yera did all the wet-bench testing of predictions. Hana Talabani provided PfPKA-C recombinant protein and Pierre Bourdoncle the image analyses. Markus Meissner advised on orthology group comparisons and Utpal Tatu and Gordon Langsley designed the study with Gordon Langsley writing the paper.

## Supplementary material

Detailed facts of importance to specialist readers are published as ”Supporting Information”. Such documents are peer-reviewed, but not copy-edited or typeset. They are made available as submitted by the authors.
