# Supplementary material for: Construction of a Plasmodium falciparum Rab-interactome identifies CK1 and PKA as Rab-effector kinases in malaria parasites
Source: Biol Cell. 2011 Nov 11;104(1):34–47. doi: 10.1111/boc.201100081 (PMC3437490; doi:10.1111/boc.201100081)
Supplement: Supplementary file 4 [file boc0104-0034-SD4.pdf]

**Legends for Supplementary Files**

**Figure S1:** Individual Ypt-interactomes, where Sfig1A shows Sec4 and Ypt1; Sfig1B shows Ypt10; Sfig1C shows Ypt11; Sfig1D shows Ypt31 and Ypt32; Sfig1E shows Vps21/Ypt51; Sfig1F, shows Ypt52; Sfig1G shows Ypt53. Red represents Rab proteins and pink indicates the interacting protein.

**Figure S2:** Homology between Ypt10, Ypt11, PfRab11B and PfRab18. Sfig.2A shows multiple sequence alignment (Clustalw) of all 4 Rabs and the respective BLASTp scores that indicate that Ypt10 is a likely PfRab18 homologue and that Ypt11 can be taken as a likely PfRab11B homologue. Sfig2B shows the very reduced interactome predicted for PfRab11B and Fig.S2C that of PfRab18.

**Figure S3: Video**
Shows a dynamic 3-D volumic reconstruction of a single cell with PfRab7 red, PfPKA-C-HA green and their co-localization white. Nuclei are stained blue with DAPI.

**Supplementary Table legends:**
**TS1:** Lists all physical and genetic interaction for Ypt proteins available at SGD.
**TS2:** Lists all predicted genetic and physical PfRab-interactants.
